# Placement of an aortomonoiliac stent graft without femorofemoral revascularization in endovascular aneurysm repair: a case report

**DOI:** 10.1186/1752-1947-5-365

**Published:** 2011-08-12

**Authors:** Michael Keese, Marco Niedergethmann, Stefan Schoenberg, Steffen Diehl

**Affiliations:** 1 Surgical Clinic, University Medical Center Mannheim, University of Heidelberg, Mannheim, Germany; 2 Clinic for Vascular and Endovascular Surgery, Klinikum der Johann Wolfgang Goethe-Universität Frankfurt, Frankfurt, Germany; 3 Department of Clinical Radiology and Nuclear Medicine, University Medical Center Mannheim, University of Heidelberg, Mannheim, Germany

## Abstract

**Introduction:**

Endovascular aortic repair, if technically feasible, is the treatment of choice for patients with a contained ruptured aortic aneurysm who are unfit for open surgery.

**Case presentation:**

We report the case of an 80-year-old Caucasian man who presented with an unusually configured, symptomatic infrarenal aortic aneurysm. His aneurysm showed an erosion of the fourth lumbar vertebra and a severely arteriosclerotic pelvic axis. A high thigh amputation of his right leg had been performed 15 months previously. On his right side, occlusion of his external iliac artery, common femoral artery, and deep femoral artery had occurred. His aneurysm was treated by a left-sided aortomonoiliac stent graft without femorofemoral revascularization, resulting in occlusions of both internal iliac arteries. No ischemic symptoms appeared, although perfusion of his right side was maintained only over epigastric collaterals.

**Conclusions:**

The placement of aortomonoiliac stent grafts for endovascular treatment of infrarenal aortic aneurysms without contralateral revascularization is a feasible treatment option in isolated cases. In this report, access problems and revascularization options in endovascular aneurysm repair are discussed.

## Introduction

The trend in the care of patients with an infrarenal aortic aneurysm is to administer endovascular treatment when feasible. This is because grade 1 evidence indicates a decrease in postoperative morbidity, a shorter hospital stay, a quicker recovery time, and a significantly lower early postoperative mortality when endovascular aneurysm repair (EVAR) is chosen as treatment [1]. However, EVAR does not improve long-term survival and has been associated with a need for continued surveillance and reinterventions at substantially increased costs. Thus, especially in high-risk patients, the emphasis has shifted toward improving patient fitness before considering any treatment of aortic aneurysms. Especially when patients present with a symptomatic aortic aneurysm or (contained) rupture, the question of whether an endovascular intervention is advisable arises. Erosion of a vertebra is considered a radiological sign of a pending rupture [2]. Survival statistics indicate a benefit for EVAR in the case of rupture [3]. If comorbidity precludes general anesthesia, EVAR is also the treatment of choice. Aortomonoiiliac prostheses extend the morphologic range of aneurysms that can be treated. The placement of an aortomonoiliac stent-graft prosthesis is also a faster and technically less complex procedure than bifurcated endovascular repair [4]. A femorofemoral bypass normally preserves perfusion of the contralateral leg. The cumulative patency rates for the femorofemoral bypass grafts for patients undergoing EVAR have generally been good [4,5]. However, this procedure is limited to patients who offer sufficient run-off to preserve graft patency.

A covered aortic stent graft is not supposed to occlude renal or internal iliac arteries [6]. Occlusion of both internal iliac arteries should generally be avoided. This is normally ensured by placement of iliac stent-graft extensions proximal to the internal iliac arteries. Although ipsilateral coil or microcoil embolization of the internal iliac artery before stent-graft extension in patients with an aortic aneurysm has been described, there is a significant risk that these patients may develop buttock claudication or even buttock gangrene [7]. In these cases, surgical revascularization of the internal iliac artery has to be performed [8]. This single-case report highlights indicatory and access-related problems in high-risk patients requiring endovascular treatment for an aortic aneurysm.

## Case presentation

A cachectic 80-year-old Caucasian man presented with lumbar pain. He had had a gastrectomy and splenectomy 20 years earlier for gastric cancer. Two years earlier, a stent had been placed in his left iliac axis for claudication occluding the origin of his internal iliac artery. Also, a high thigh amputation had been performed on his right leg 15 months earlier. An infrarenal aortic aneurysm with a maximum diameter of 3.5 cm had been diagnosed 12 months earlier. He also presented with artrial fibrillation, severe emphysema, and a history of stroke with an incomplete left hemiparesis.

**Figure 1:**
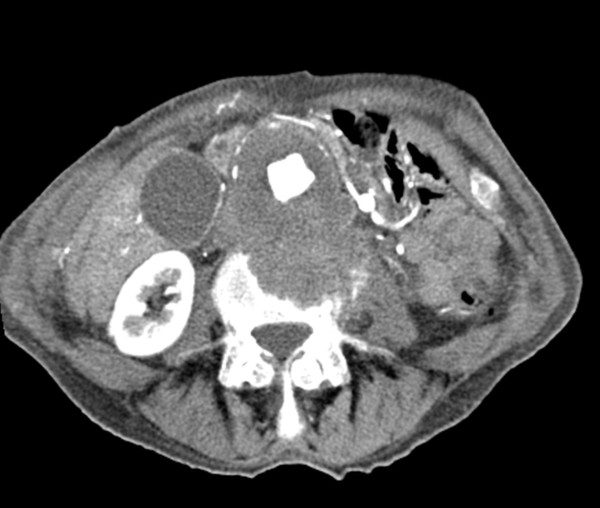
**Arterial phase computed tomography (CT) image of the erosion of the fourth lumbar vertebra**.

**Figure 2:**
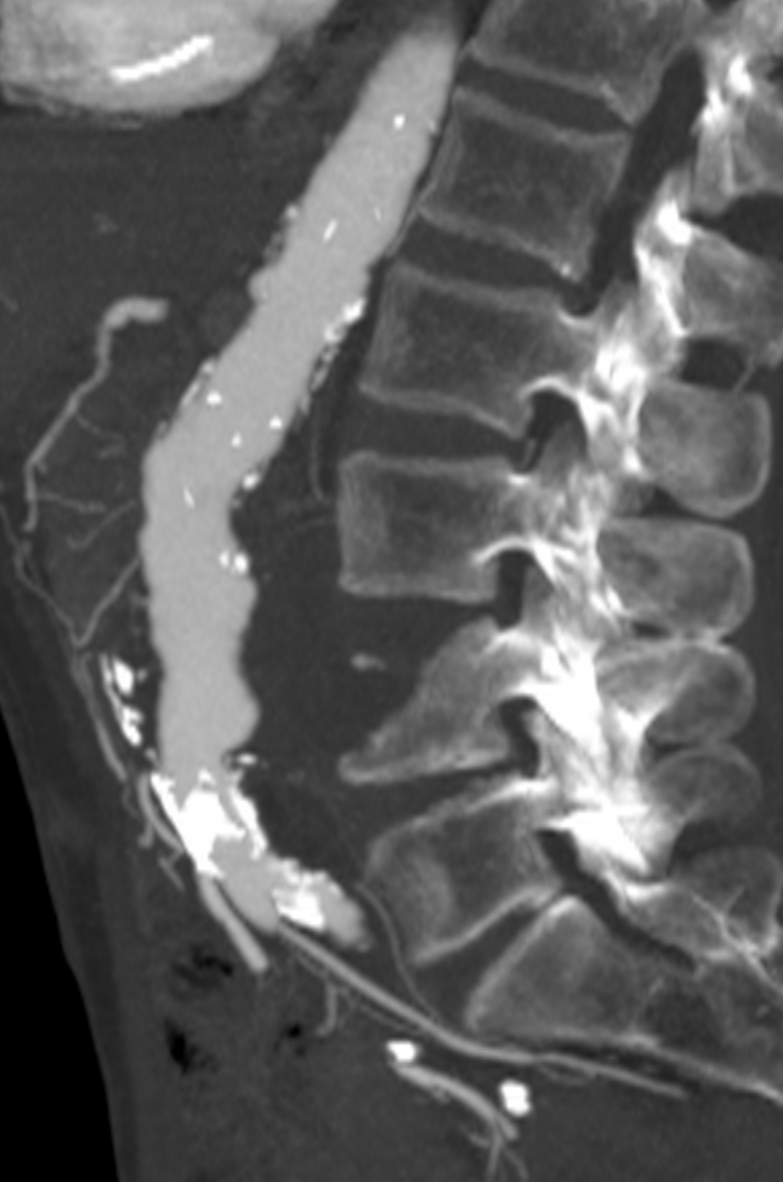
**Maximum-intensity sagittal reconstruction showing the craniocaudal extension of the aortic aneurysm**.

**Figure 3:**
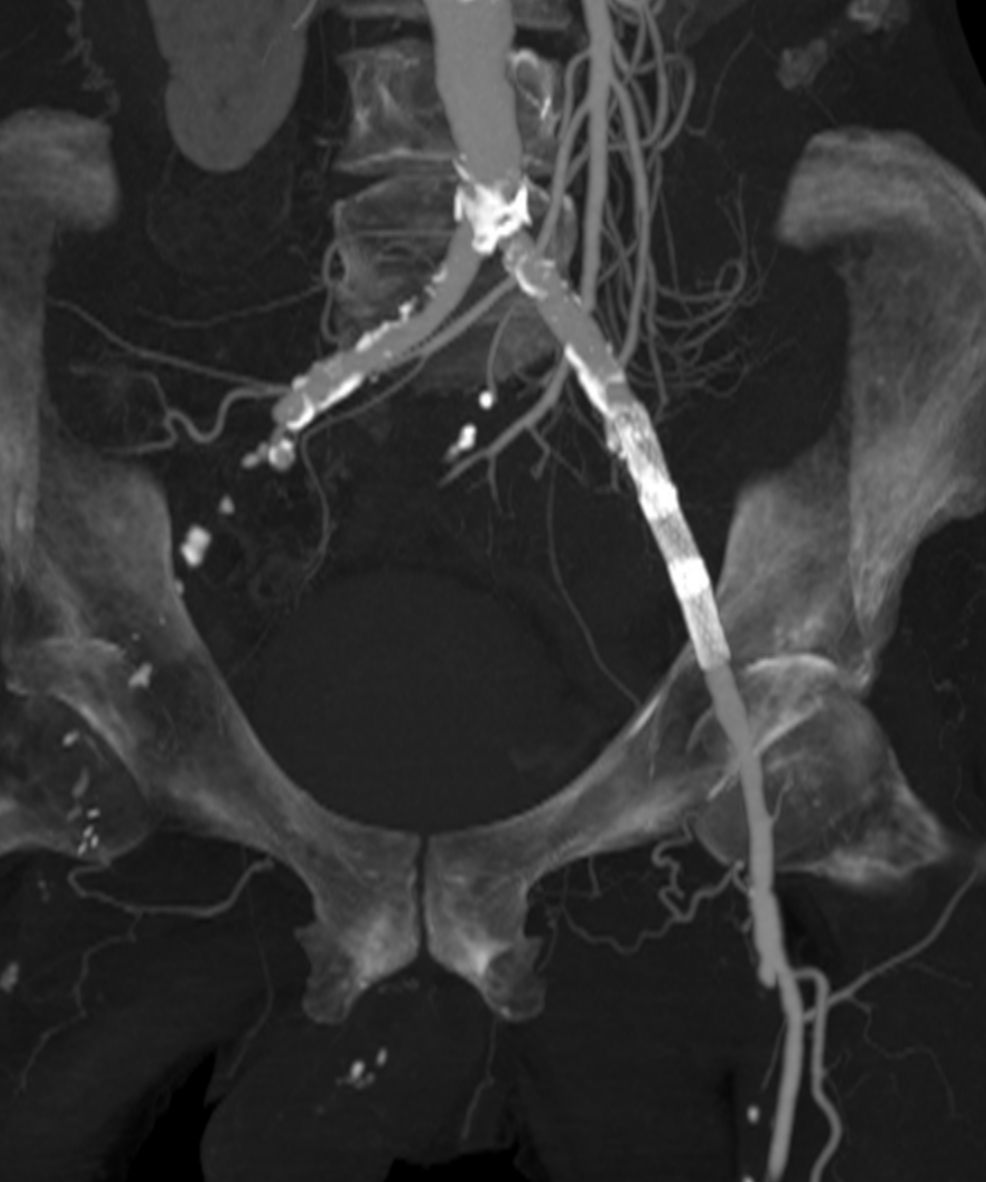
**Maximum-intensity reconstruction of the pelvic axis with occlusion of the right external artery and the stent, which was formally placed over the left hypogastric artery**.

A computed tomography (CT) examination revealed a 5.9×5 cm infrarenal aortic aneurysm with destruction of his fourth lumbar vertebra. No extravasation of contrast agent was detected (Figures 1, 2, 3). His aneurysm started 0.9 cm below his renal arteries and extended into his aortic bifurcation. The examination also revealed an occlusion of his right external iliac artery and severe calcifications of his left pelvic axis. Our patient was considered to be at high risk for any procedure requiring general anesthesia.

**Figure 4:**
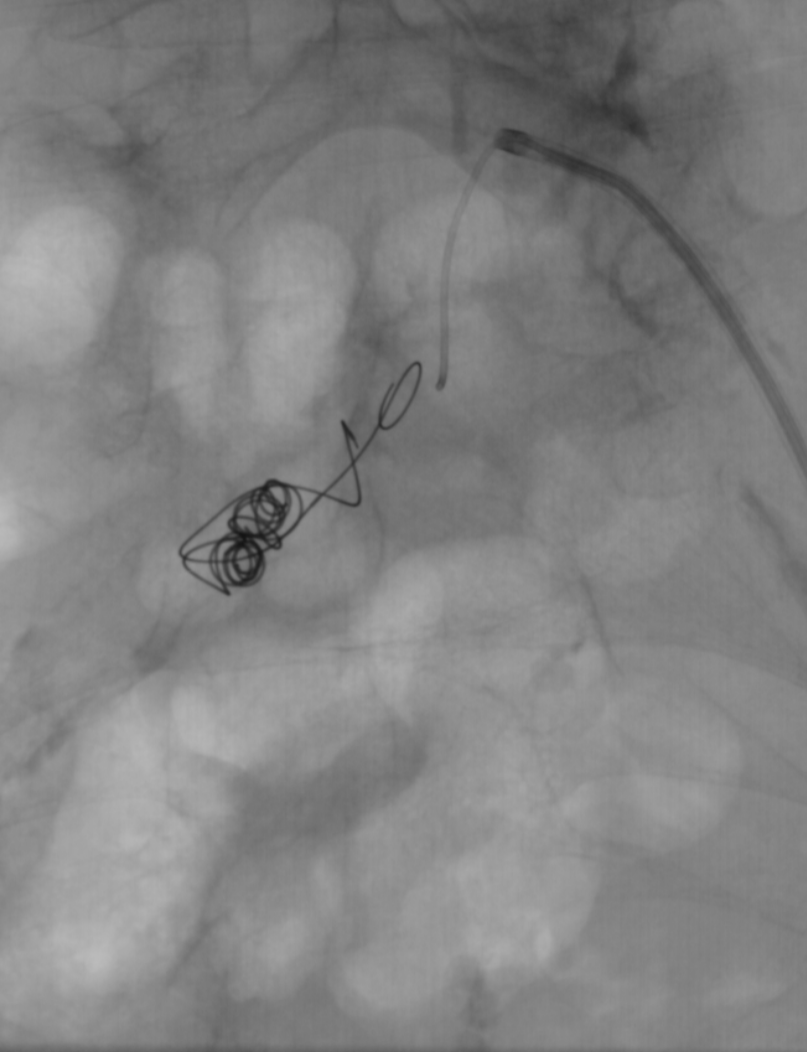
**Occlusion of the right common iliac artery with microcoils**.

**Figure 5:**
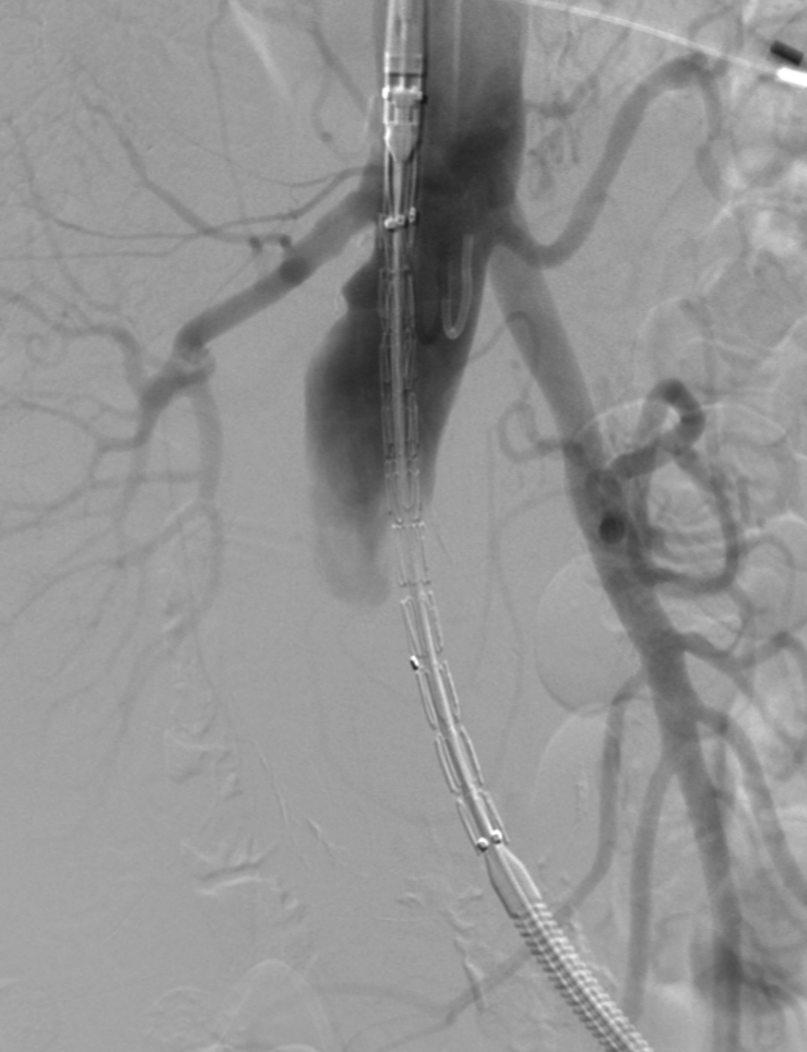
**Advancement of the aortic stent graft (Endurant ENUF2514C105EE; Medtronic, Minneapolis, MN, USA)**.

**Figure 6:**
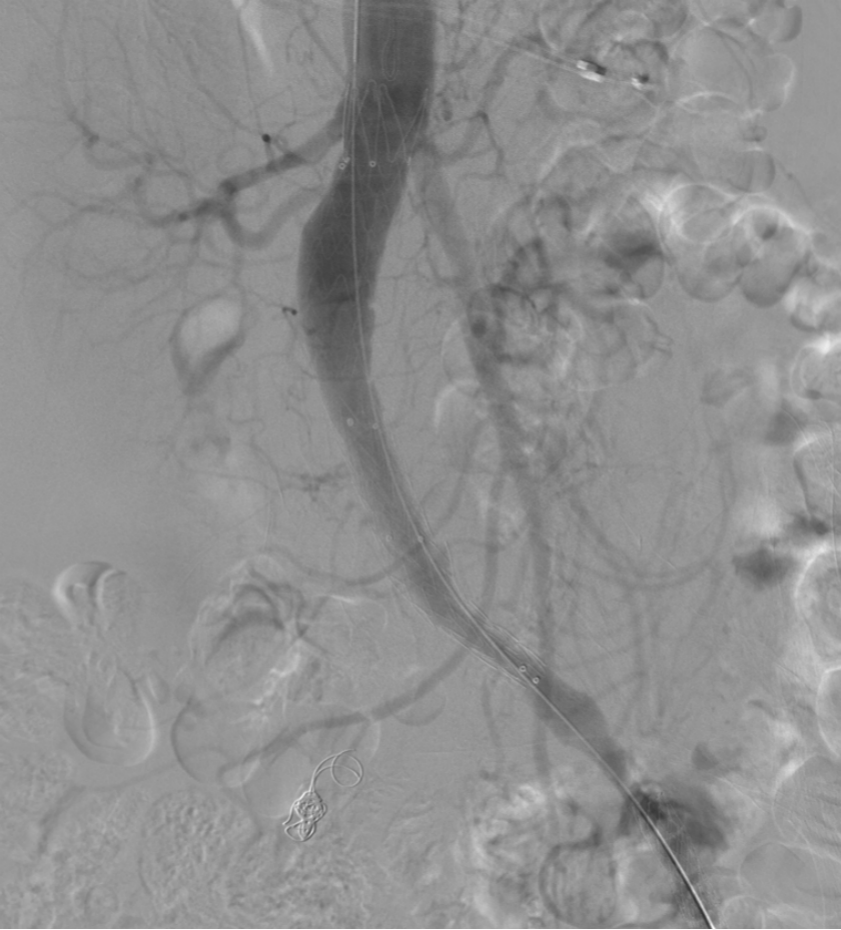
**Angiography image of infrarenal placement of the stent graft**.

**Figure 7:**
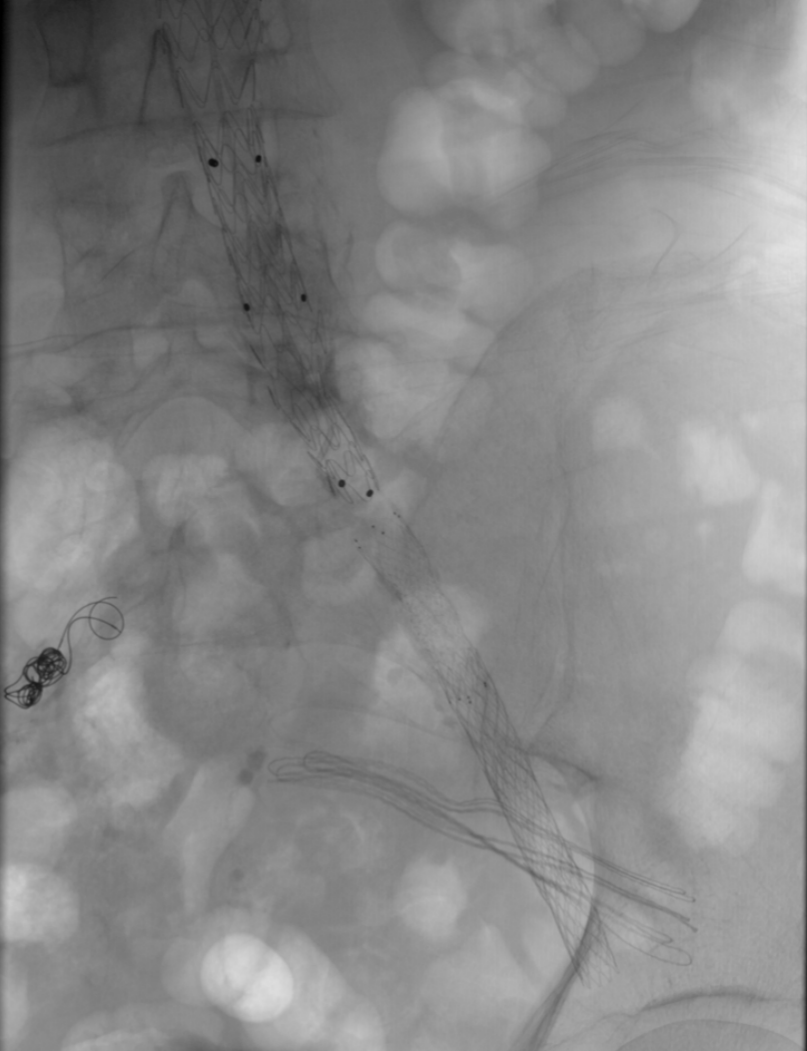
**X-ray of the completed reconstruction with stent graft, leg extension, and microcoils**.

The clinical history of back pain and the erosion of his lumbar vertebra were suspicious of a pending rupture of the aneurysm. Considering the multiple surgical risk factors, we chose an endovascular treatment. The long, calcified occlusion of his right external iliac artery and his right common femoral artery could not be recanalized to place an aortic-biiliac stent graft. Therefore, his right common iliac artery had to be embolized to prevent endoleakage, and an occlusion of the formerly patent proximal portion of his remnant right internal iliac artery had to be accepted. This approach was chosen since collaterals over the epigastric artery were considered sufficient to preserve the viability of the right stump. After crossover access was gained, coils were placed via a microcatheter. The aortic stent graft and the iliac leg extension (Endurant ENUF2514C105EE, ENLW1613C95EE^®^; Medtronic, Minneapolis, MN, USA) were advanced via our patient's left pelvic axis, successfully excluding the aneurysm. A control angiography revealed no endoleakage. A short dissection of his left external iliac artery was treated by an uncovered balloon-expandable stent (Figures 4, 5, 6, 7).

After this intervention, his back pain resolved. A CT-guided biopsy of his fourth lumbar vertebra revealed a hematoma, and an inflammatory process could be ruled out. CT also confirmed a correct placement of the stent graft without endoleakage. Our patient developed no signs of gluteal ischemia and was discharged two days later. He was well when last seen three months later.

## Conclusions

Endovascular exclusion of an infrarenal aortic aneurysm may be performed using aortobiiliac or aortomonoiliac stent grafts. The applicability of aortic stent grafts is limited by accessibility of the pelvic axis. Also, both perfusion of the two internal iliac arteries and - if aortomonoiliac stent grafts are placed - perfusion of the contralateral leg have to be considered. This case report describes a patient with an unusually configured infrarenal aortic aneurysm with a difficult vascular anatomy. Also, EVAR is a standard procedure for the treatment of aortic aneurysms [1,6]. To the best of our knowledge, no similar case, in which only an aortomonoiliac prothesis without any contralateral revascularization was placed, has been reported. Our case, therefore, highlights that the endovascular treatment of an infrarenal aortic aneurysm has to be adjusted to the clinical requirements and vascular anatomy of each patient.

## Abbreviations

CT: computed tomography; EVAR: endovascular aneurysm repair.

## Consent

Written informed consent was obtained from the patient for publication of this case report and any accompanying images. A copy of the written consent is available for review by the Editor-in-Chief of this journal.

## Competing interests

The authors declare that they have no competing interests.

## Authors' contributions

MK and SD performed the endovascular procedure. MN and SS provided technical advice. All authors have read and approved the final manuscript.
